# Early environmental predictors for attention-deficit hyperactivity disorder (ADHD), autism spectrum disorder (ASD) and their co-occurrence: The prospective ABIS-Study

**DOI:** 10.1038/s41598-024-65067-4

**Published:** 2024-06-26

**Authors:** Andrea Lebeña, Åshild Faresjö, Michael P. Jones, Felicia Bengtsson, Tomas Faresjö, Johnny Ludvigsson

**Affiliations:** 1 Division of Pediatrics, Department of Biomedical and Clinical Sciences, Linköping University, US Campus, Building 511 (14, 09B), 581 83 Linköping, Sweden; 2 Department of Medicine and Health, Community Medicine, Linköping University, Linköping, Sweden; 3 School of Psychological Sciences, Macquarie University, North Ryde, NSW Australia; 4 Crown Princess Victoria Children’s Hospital, Region Östergötland, Linköping, Sweden

**Keywords:** ADHD, ASD, Co-occurrence, Etiology, Environmental exposure, Psychosocial vulnerability, Epidemiology, ABIS, Psychology, Environmental social sciences, Risk factors

## Abstract

ADHD and ASD are highly heritable and show a high co-occurrence and persistence into adulthood. This study aimed to identify pre and perinatal risk factors, and early psychosocial exposures related to later diagnosis of ADHD, ASD, and their co-occurrence. 16,365 children born 1997–1999 and their families, involved in the prospective population-based ABIS study (All Babies in Southeast Sweden), were included in this sub-study. Pre and perinatal factors and early environmental psychosocial exposures were collected from parental-questionnaires at birth and 1-year follow-up. Diagnoses from birth up to 23 years of age were obtained from the Swedish National Diagnosis Register in 2020. The cumulative incidence of ADHD, ASD, and their co-occurrence in the ABIS-cohort Study were 4.6%, 1.7%, and 1.1%, respectively. Being male was associated with an increased risk for ADHD, ASD, and their co-occurrence (aOR 1.30, 1.56, and 1.91, respectively), while higher household income reduced it (aOR 0.82, 0.73, and 0.64). Serious life events during pregnancy (aOR 1.40) and maternal smoking (aOR 1.51) increased the risk of ADHD, while older maternal age (aOR 0.96), higher parental education (aOR 0.72 maternal and aOR 0.74 paternal) and longer exclusive breastfeeding (aOR 0.72) reduced it. Non-Swedish paternal nationality (aOR 0.40) and higher maternal education (aOR 0.74) were associated with a lower risk of ASD, while a family history of autoimmune diseases increased the risk of the co-occurrence of both disorders (aOR 1.62). Obtained results suggest that the etiology of ADHD, ASD, and their co-occurrence is independently associated with environmental psychosocial predictors. The co-occurrence seems to overlap the etiology of ADHD, in which psychosocial determinants have a larger role, however, it is also independently influenced by a family history of autoimmune diseases.

## Introduction

Attention deficit hyperactivity disorder (ADHD) and autism spectrum disorder (ASD) are the neurodevelopmental disorders (NDDs) most commonly diagnosed, with prevalence estimates of 5–10% and 1–2%, respectively^1,2^. Their onset occurs in childhood and is associated with significant behavioral, academic, emotional, and adaptive problems^3^. Both disorders have been considered lifelong conditions and show a substantial degree of persistence into adulthood.

Males exhibit NDDs more often than females, however, the mechanisms that lead to this sexual dimorphism are only partly understood^4^. NDDs are considered highly heritable and have multiple and complex causes, including both genetic and environmental factors^5–7^. The etiology of ADHD includes preterm birth, low birth weight, maternal stress, alcohol use and smoking, and severe early deprivation among others^8,9^. Evidence suggested that low birth weight, birth complications, advanced parental age, maternal diseases, and both bacterial and viral infections, increased the risk of ASD^10,11^. Toxic chemical exposure such as air pollutants, heavy metals, and pesticides, were also studied^12^. It was suggested that prenatal multivitamins supplementation decreased the risk of ASD and ADHD^13,14^, but also longer breastfeeding was associated with a lower risk of ASD and ADHD and better cognitive development^15^.

While the most studied etiological factors were primarily biological, psychosocial exposures, also recognized as autism and ADHD determinants by The WHO’s interactive biopsychosocial model (The International Classification of Functioning—ICF), have received less attention^16^. Despite the evidence that suggests that ADHD and ASD are heterogeneous in etiology, biology, presentation, and associated functional impairment^17,18^, it was estimated that between 30 and 50% of individuals with ASD manifest ADHD symptoms, similarly, two-thirds of individuals with ADHD show features of ASD^19^. The high co-occurrence rates between these two disorders suggests that they might share developmental pathways and risk factors^4^, most research to date has considered separate developmental trajectories, therefore little is known regarding the etiology of their co-occurrence^20^.

The prospective longitudinal design of the ABIS-Study that includes a large representative sample with more than 22 years of follow-up, together with the validated register data for ADHD and ASD diagnoses, that made the loss to follow-up minimal, offers the opportunity to determine whether the two disorders share developmental trajectories and early risk factors.

The current study aimed to examine pre and perinatal risk factors and early environmental psychosocial exposures, that could increase the long-term risk of developing ADHD, ASD, and the co-occurrence of both disorders.

## Methods

### Study population

This prospective cohort study includes data from the ABIS-Study (All Babies in Southeast Sweden), a longitudinal, population-based cohort study based on data collected from 16,365 children born between October 1997 and October 1999 in Southeast Sweden and their respective families. ABIS-Study aims to investigate how environmental and genetic factors influence the development of immune-mediated diseases, which then includes ADHD and ASD, where immune mechanisms may play a role^10^. The children included in the ABIS-Study have been followed from birth onwards, and questionnaire data, biological samples, and register data have been collected at birth and age of 1, 3, 5, 8, 10–12, 17–19, and 23–25 years.

The parents were given both oral and written information before giving informed consent to participate in the study. The ABIS-study was conducted according to the Helsinki Declaration and was approved by the Research Ethics Committees at Linköping University (Dnr: LIU 287-96, LIU 321-99, LIU 2011/53-32 and LIU 2003-092), and Lund University (LU 83-97) in Sweden. Connection of the ABIS registers to National Registers was approved by the Research Ethics Committee in Linköping (Dnr 2013/253-32). All data used in this study were also anonymized.

### Diagnoses of ADHD, ASD, and their co-occurrence

**Figure 1:**
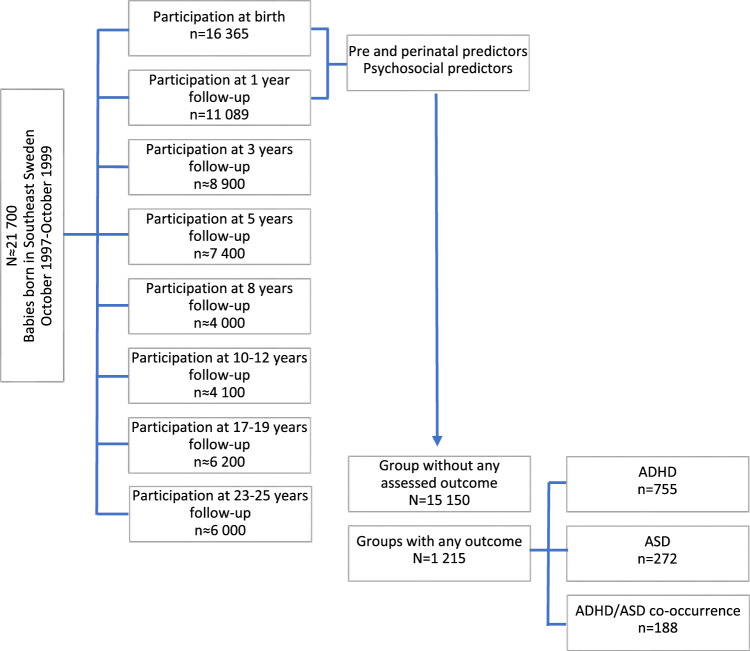
Study population flow-chart. Definition of groups based on the cumulative incidence rates for ADHD, ASD, and their co-occurrence from birth until the age of 23 years. *ADHD* attention-deficit/hyperactivity disorder, *ASD* autism spectrum disorder.

The diagnoses of ADHD and ASD were obtained for the entire ABIS population (n = 16,365) from birth until 2020-12-31, by cross-linking with the Swedish National Patient Register (NPR), containing all hospital inpatients (since 1973) and outpatients (since 2001) International Classification of Diseases (ICD-8 to ICD-10) based on doctor-set diagnoses^21^. According to the ICD-10, F90 (F90.0, F90.1, F90.8, and F90.9) and F84 (F84.0, F84.1, F84.2, F84.3, F84.4, F84.5, F84.8, and F84.9) were the diagnostic codes used for ADHD and ASD, respectively. Those children that got a unique diagnosis of ADHD (n = 755), those that received a unique ASD diagnosis (n = 272), and those that got both diagnoses (ADHD and ASD co-occurrence) (n = 188), according to the NPR, were defined as the groups any outcome, the rest of the study population constituted the group without any assessed outcome (n = 15,150) (Fig. 1).

### Predictors

Data was collected from the questionnaires used in the ABIS-Study at birth and 1-year follow-up.

#### Pre and perinatal

Maternal disease during pregnancy was a composite measure including hypothyroidism and hyperthyroidism, B12 deficiency, systemic lupus erythematosus, adrenal insufficiency, type 1 and type 2 diabetes, gestational diabetes, celiac disease, rheumatism, and inflammatory bowel disease. Autoimmune disease heredity was dichotomized as no autoimmune heredity or at least one first-degree relative with any of the autoimmune diseases previously mentioned^22^. Infections during pregnancy, and iron and vitamins/minerals supplements use during pregnancy, were dichotomic variables. Parity was categorized in “first parity” or “previous parity”, twins (single fetus or multiple fetuses), way of delivery (vaginal, cesarean section, or other complications), gestational age, and birth weight, were also included.

#### Psychosocial

Child’s sex, maternal and paternal age at child’s birth, maternal and paternal ethnicity (born in Sweden or abroad), civil status at child’s birth (married/living with a partner or single parent), support during pregnancy, and security for mother and child were also included. Serious life events during pregnancy and at 1 year of age^23^, maternal smoking, and tobacco exposure at 1 year of age, were also included. Exclusive breastfeeding duration was categorized as “less than 4 months”, “5–8 months”, and “9 months or more”, according to WHO recommendations^24^. Maternal and paternal education was classified according to the International Standard Classification of Education (ISCED) and graded in three levels: low = ISCED level I–II, medium = ISCED level III–IV, and high = ISCED level V–VII^25^. The disposable household income for the year 2000 was obtained from the Swedish Income and Tax register and was categorized into three levels based on percentage distribution, low income (bottom quintile), medium income (second to fourth quintile), and high income (top quintile)^26^.

### Statistical methods

All statistical analyses were performed in SPSS software version 28.0 (IBM SPSS Inc., Chicago, IL, USA). Dichotomous predictors were presented as frequencies and percentages, and differences were assessed using Chi-squared test, while quantitative predictors were described using mean and standard deviation, and differences between groups were assessed using *t*-test. A *P*-value ≤ 0.05 was considered statistically significant, and multiple comparisons between the groups with any assessed outcome were adjusted using Bonferroni correction (Table 1). Missing values were imputed via multiple imputation in which all predictor and outcome variables were employed in the imputation process which used 5 imputation samples^27^. All reported univariate and multiple multinomial logistic regression analyses were fitted to the data after multiple imputation. Identification of statistically independent discriminators used a backward elimination algorithm in which all univariately statistically significant discriminators (unadjusted model—Table 2) were entered into a single full model. Individual discriminators were then removed from the model in a stepwise fashion if their individual *P*-value was > 0.05 until all remaining discriminators were statistically significant (Multivariable model of independent predictors—Table 2). Automatic model selection algorithms are known to be prone to over-optimism^28^ and hence we regard the final model selected as a candidate model which is in need of independent replication. Effect sizes were reported as odds ratios (OR) or adjusted odds ratios (aOR) with 95% confidence intervals (95% CI) and 2-tailed *P*-values. We conducted a response-bias analysis.

## Results

The cumulative incidence rates for ADHD, ASD, and their co-occurrence in ABIS Study were 4.6%, 1.7%, and 1.1%, respectively, and the prevalence was higher in boys (*P* < 0.001). The median (and interquartile range) of the age at first diagnosis were 16 (6) for ADHD, 17 (6) for ASD, and 13 (6) for the co-occurrence. A total of 66 children from the initial ABIS cohort died before 23 years of age, the average age (and SD) was 14.4 (7.8).

### ADHD predictors

The ADHD group was characterized by a higher prevalence of family history of autoimmune diseases (*P* < 0.001), a higher proportion of preterm babies (gestational age below 37 weeks) (*P* = 0.019), and lower birth weight (*P* = 0.024). Concerning psychosocial factors, the ADHD group was characterized by younger parents (below 25 years of age, *P* < 0.001), low parental education (*P* < 0.001), higher proportion of single parents (*P* < 0.001), low household income (*P* < 0.001), higher prevalence of maternal smoking (*P* < 0.001), and tobacco exposure at 1 year of age (*P* < 0.001). The ADHD group also exhibited a higher proportion of serious life events during pregnancy (*P* < 0.001), and shorter breastfeeding duration (less than 4 months, *P* < 0.001) compared to the controls, but also to the ASD group (Table 1).

**Table 1:** Distribution of potential predictors of ADHD, ASD, and their co-occurrence among the groups with any outcome and the one with none.

	ADHD^a^ (*n* = 755)	*P* value	ASD^b^ (*n* = 272)	*P* value	Co-occurrence^c^ (*n* = 188)	*P* value	Group without any assessed outcome (*n* = 15,150)	Multiple comparisons among the groups with any outcome
Pre and perinatal predictors								
AD heredity		**< 0.001**		0.535		**< 0.001**		
Yes	140 (18.5%)		46 (16.9%)		45 (23.9%)		2477 (16.3%)	
No	615 (81.5%)		226 (83.1%)		143 (76.1%)		12,706 (83.7%)	
Maternal diseases		0.057		**0.045**		0.322		
Yes	60 (7.9%)		25 (9.2%)		15 (8.0%)		944 (6.2%)	
No	695 (92.1%)		247 (90.8%)		173 (92.0%)		14,235 (93.8%)	
Infections (pregnancy)		0.504		**0.019**		0.403		
Yes	247 (34.0%)		102 (39.7%)		65 (35.7%)		4821 (32.8%)	
No	480 (66.0%)		155 (60.3%)		117 (64.3%)		9885 (67.2%)	
Iron supplements (pregnancy)		0.967		0.614		0.891		
Yes	600 (82.4%)		211 (81.2%)		150 (87.0%)		12,067 (82.4)	
No	128 (17.6)		49 (18.8%)		33 (18.0%)		2585 (17.6%)	
Vitamins/minerals supplements (pregnancy)		0.386		0.536		0.529		
Yes	415 (57.8%)		151 (58.1%)		103 (58.5%)		8147 (56.2%)	
No	303 (42.2%)		109 (41.9%)		73 (41.5%)		6361 (43.8%)	
Parity		0.802		0.476		0.847		
First parity	297 (40.1%)		115 (42.8%)		76 (41.3%)		6001 (40.6%)	
Previous parity	443 (59.9%)		154 (57.2%)		108 (58.7%)		8780 (59.4%)	
Twins		0.744		0.592		0.251		
Single fetus	736 (97.5%)		267 (98.2%)		186 (98.9%)		14,825 (97.7%)	
Multiple fetuses	19 (2.5%)		5 (1.8%)		2 (1.1%)		354 (2.3%)	
Way of delivery		0.213		**0.017**		0.331		**b,c**
Normal	621 (84.8%)		225 (86.5%)		144 (78.7%)		12,160 (82.9%)	
C-section	83 (11.3%)		17 (6.5%)		27 (14.8%)		1754 (12%)	
Other complication	28 (3.8%)		18 (6.9%)		12 (6.6%)		762 (5.2%)	
Gestational age^#^		**0.019**		0.703		**0.027**		
Preterm (< 37 weeks)	45 (6.1%)		10 (3.8%)		14 (7.6%)		635 (4.3%)	
In term	695 (93.9%)		253 (96.2%)		170 (92.4%)		14,196 (95.7%)	
Birth weight mean (SD)	3.53 (0.56)	**0.024**	3.60 (0.52)	0.579	3.58 (0.61)	0.897	3.58 (0.56)	
Psychosocial predictors								
Sex		**< 0.001**		**< 0.001**		**< 0.001**		
Boy	437 (57.9%)		169 (62.1%)		126 (67.0%)		7753 (51.2%)	
Girl	318 (42.1%)		103 (37.9%)		62 (33.0%)		7397 (48.8%)	
Maternal age^#^		**< 0.001**		**0.005**		0.084		**a,b**
< 25	261 (34.7%)		73 (26.9%)		51 (27.1%)		3184 (21.2%)	
26–35	443 (58.9%)		164 (60.5%)		117 (62.2%)		10,471 (69.6%)	
> 36	48 (6.4%)		34 (12.5%)		20 (10.6%)		1391 (9.2%)	
Paternal age^#^		**< 0.001**		0.194		0.082		
< 25	135 (18.5%)		36 (13.6%)		28 (15.2%)		1505 (10.2%)	
26–35	483 (66.2%)		178 (67.2%)		121 (65.8%)		10,252 (69.4%)	
> 36	112 (15.3%)		51 (19.2%)		35 (19.0%)		3010 (20.4%)	
Maternal education		**< 0.001**		**< 0.001**		**< 0.001**		**a,b**
Low	135 (18.4%)		38 (14.4%)		29 (15.7%)		1176 (8.0%)	
Medium	462 (63.1%)		151 (57.4%)		115 (62.2%)		8797 (59.5%)	
High	135 (18.4%)		74 (28.1%)		41 (22.2%)		4819 (32.6%)	
Paternal education		**< 0.001**		0.374		**< 0.001**		**a,b**
Low	173 (24.0%)		41 (16.0%)		36 (19.7%)		1892 (13.0%)	
Medium	442 (61.2%)		154 (59.9%)		119 (65.0%)		9011 (61.8%)	
High	107 (14.8%)		62 (24.1%)		28 (15.3%)		3668 (25.2%)	
Maternal ethnicity		0.309		0.184		0.498		
Swedish	695 (94.3%)		241 (91.3%)		175 (94.6%)		13,850 (93.3%)	
Other	42 (5.7%)		23 (8.7%)		10 (5.4%)		987 (6.7%)	
Paternal ethnicity		0.321		0.114		0.693		
Swedish	670 (91.8%)		252 (96.6%)		173 (93.5%)		13,731 (92.8%)	
Other	60 (8.2%)		9 (3.4%)		12 (6.5%)		1072 (7.2%)	
Civil status		**< 0.001**		0.084		**< 0.001**		
Living with partner	701 (95.1%)		255 (96.6%)		175 (94.6%)		14,532 (98.1%)	
Single parent	36 (4.9%)		9 (3.4%)		10 (5.4%)		285 (1.9%)	
Household income		**< 0.001**		**< 0.001**		**< 0.001**		
Low	228 (30.4%)		75 (27.8%)		61 (32.4%)		2878 (19.2%)	
Medium	429 (57.2%)		148 (54.8%)		106 (56.4%)		9046 (60.3%)	
High	93 (12.4%)		47 (17.4%)		21 (11.2%)		3081 (20.5%)	
Support (pregnancy)		0.124		0.513		**0.002**		
Yes	720 (98.8%)		260 (99.6%)		177 (97.3%)		14,560 (99.3%)	
No	9 (1.2%)		1 (0.4%)		5 (2.7%)		107 (0.7%)	
Security (pregnancy)		0.054		0.964		**< 0.001**		
Yes	720 (99.2%)		258 (99.6%)		179 (97.8%)		14,575 (99.6%)	
No	6 (0.8%)		1 (0.4%)		4 (2.2%)		54 (0.4%)	
Serious life event (pregnancy)		**< 0.001**		0.408		0.198		**a,b**
Yes	102 (13.9%)		20 (7.6%)		25 (13.7%)		1339 (9.1%)	
No	631 (86.1%)		243 (92.4%)		158 (86.3%)		13,403 (90.9%)	
Serious life event (1 year)		0.156		0.154		**0.002**		
Yes	27 (6.3%)		10 (6.3%)		11 (9.8%)		400 (4.1%)	
No	405 (93.8%)		148 (93.7%)		101 (90.2%)		9449 (95.9%)	
Maternal smoking		**< 0.001**		0.210		**< 0.001**		
Yes	161 (22.0%)		38 (14.4%)		36 (19.6%)		1544 (10.4%)	
No	572 (78.0%)		225 (85.6%)		148 (80.4%)		13,253 (89.6%)	
Tobacco exposure (1 year)		**< 0.001**		0.566		0.522		
Yes	51 (11.3%)		11 (6.5%)		5 (4.1%)		559 (5.5%)	
No	402 (88.7%)		159 (93.5%)		116 (95.9%)		9676 (94.5%)	
Breastfeeding duration		**< 0.001**		0.583		0.186		**a,b**
< 4 months	131 (30.3%)		24 (15.0%)		28 (25.2%)		1580 (16%)	
5–8 months	159 (36.7%)		59 (36.9%)		41 (36.9%)		3935 (39.9%)	
> 9 months	143 (33.0%)		77 (48.1%)		42 (37.8%)		4337 (44%)	

*ADHD* attention-deficit/hyperactivity disorder, *ASD* autism spectrum disorder, *AD* autoimmune disease. Values are presented as mean (SD) or as absolute numbers (percentages). # Continuous variables categorized only for descriptive purposes. All *P*-values were calculated for each group with any outcome against the one without any assessed outcome from Chi-squared test and *t*-test. The multiple testing correction threshold was *P* < 0.016. The last column indicates the statistically significant differences found between the groups with any outcome (a: ADHD, b: ASD, c: co-occurrence), and its magnitude. Significant values are in bold.

### Statistically independent predictors of ADHD

Being male, lower household income, and lower maternal education, together with younger mother, maternal smoking, serious life event during pregnancy, lower paternal education, and short breastfeeding duration, were the risk factors that remain significant in the multiple multinomial logistic regression analyses (Table 2).

**Table 2:** Multinomial logistic regression analysis, effect sizes represented as odds ratios. Groups with any outcome compared to the one with none.

	Unadjusted model^a^			Multivariable model of independent predictors^b^		
	ADHD (*n* = 755) OR (95% CI)	ASD (*n* = 272) OR (95% CI)	Co-occurrence (*n* = 188) OR (95% CI)	ADHD (*n* = 755) aOR (95% CI)	ASD (*n* = 272) aOR (95% CI)	Co-occurrence (n = 188) aOR (95% CI)
Pre and perinatal predictors						
AD heredity	1.17 (0.97–1.41)	1.04 (0.76–1.44)	1.61 (1.15–2.26)**	1.20 (0.99–1.45)	1.04 (0.75–1.43)	1.62 (1.15–2.28)**
Maternal diseases	1.30 (0.99–1.71)	1.52 (1.01–2.31)*	1.31 (0.77–2.22)			
Infections (pregnancy)	1.06 (0.90–1.25)	1.33 (1.04–1.72)*	1.11 (0.82–1.51)			
No iron intake (pregnancy)	1.00 (0.82–1.22)	1.07 (0.78–1.47)	1.02 (0.69–1.51)			
No vitamins/minerals intake (pregnancy)	0.94 (0.81–1.09)	0.93 (0.72–1.20)	0.91 (0.67–1.24)			
Previous parity	1.03 (0.88–1.19)	0.91 (0.71–1.16)	0.96 (0.71–1.29)			
Multiple fetuses	1.08 (0.67–1.72)	0.78 (0.32–1.91)	0.45 (0.11–1.82)			
C-section^#^	0.92 (0.73–1.16)	0.56 (0.34–0.92)*	1.30 (0.87–1.96)			
Other delivery complication^#^	0.74 (0.50–1.09)	1.24 (0.76–2.01)	1.29 (0.72–2.34)			
Gestational age (weeks)	0.95 (0.91–0.99)**	1.01 (0.94–1.09)	0.94 (0.87–1.01)			
Birth weight (g)	0.86 (0.75–0.98)*	1.07 (0.86–1.33)	1.03 (0.79–1.33)			
Psychosocial predictors						
Sex (boy)	1.31 (1.13–1.52)***	1.56 (1.22–2.00)***	1.94 (1.43–2.63)***	1.30 (1.12–1.51)***	1.56 (1.22–2.00)***	1.91 (1.41–2.60)***
Maternal age (years)	0.93 (0.92–0.95)***	1.00 (0.97–1.02)	0.97 (0.94–1.00)	0.96 (0.95–0.98)***	1.01 (0.99–1.04)	1.00 (0.97–1.03)
Paternal age (years)	0.95 (0.94–0.97)***	1.00 (0.98–1.02)	0.98 (0.95–1.00)			
Maternal education	0.49 (0.43–0.55)***	0.73 (0.59–0.90)**	0.60 (0.47–0.76)***	0.72 (0.63–0.84)***	0.74 (0.59–0.93)**	0.79 (0.59–1.05)
Paternal education	0.55 (0.49–0.63)***	0.88 (0.72–1.07)	0.63 (0.50–0.81)***	0.74 (0.64–0.85)***	1.05 (0.84–1.32)	0.83 (0.63–1.08)
Non-Swedish mother	0.84 (0.61–1.16)	1.30 (0.85–2.01)	0.78 (0.41–1.49)			
Non-Swedish father	1.17 (0.89–1.53)	0.44 (0.23–0.87)*	0.87 (0.48–1.56)	0.96 (0.73–1.26)	0.40 (0.20–0.78)**	0.73 (0.40–1.32)
Single parent	2.63 (1.85–3.75)***	1.80 (0.92–3.54)	2.89 (1.51–5.52)***			
Household income	0.61 (0.54–0.69)***	0.73 (0.61–0.89)**	0.56 (0.45–0.71)***	0.82 (0.72–0.94)**	0.73 (0.60–0.90)**	0.64 (0.50–0.82)***
Lack of support (pregnancy)	1.74 (0.88–3.47)	0.49 (0.07–3.53)	3.77 (1.52–9.37)**			
Lack of security (pregnancy)	2.22 (0.94–5.22)	0.93 (0.13–6.78)	5.51 (1.98–15.35)***			
Serious life event (pregnancy)	1.60 (1.29–1.99)***	0.82 (0.52–1.30)	1.55 (1.01–2.37)*	1.40 (1.13–1.75)**	0.79 (0.49–1.26)	1.38 (0.90–2.13)
Serious life event (1 year)	1.39 (0.96–2.03)	1.46 (0.68–3.13)	1.95 (1.02–3.75)*			
Maternal smoking	2.33 (1.94–2.79)***	1.40 (0.99–1.99)	2.12 (1.48–3.05)***	1.51 (1.24–1.85)***	1.29 (0.88–1.88)	1.42 (0.96–2.12)
Tobacco exposure (1 year)	2.00 (1.56–2.57)***	1.15 (0.59–2.25)	1.31 (0.50–3.46)			
Breastfeeding duration	0.71 (0.64–0.79)***	1.12 (0.88–1.43)	0.81 (0.66–0.99)*	0.72 (0.63–0.83)***	1.13 (0.83–1.54)	0.81 (0.63–1.03)

*ADHD* attention-deficit/hyperactivity disorder, *ASD* autism spectrum disorder, *AD* autoimmune disease, *OR* odds ratio, *aOR* adjusted odds ratio.

^a^Unadjusted independent variables for ADHD (n = 755), ASD (n = 272), the co-occurrence of both disorders (n = 188), and 15.150 subjects without any assessed outcome.

^b^Multivariable model of independent predictors.

^#^Vaginal delivery acts as the reference category.

*P < 0.05, **P < 0.01 and ***P < 0.001.

### ASD predictors

Maternal diseases (*P* = 0.045) and infections during pregnancy (*P* = 0.019) were more frequently reported by the ASD group. Cesarean sections (*P* = 0.017) were reported to a lesser extent than controls and the co-occurrence group. The ASD group was also significantly associated with older mothers (above 36 years of age) compared to controls and the ADHD group. Though the ASD group showed a higher proportion of highly educated mothers and fathers than the ADHD group, they exhibited more often low maternal education level (*P* < 0.001), and low household income (*P* < 0.001) than the control group (Table 1).

### Statistically independent predictors of ASD

Being male, lower household income, and lower maternal education together with paternal Swedish nationality, were the risk factors that remain significant in the multiple multinomial logistic regression analyses (Table 2).

### ADHD and ASD co-occurrence predictors

The co-occurrence group was characterized by a higher prevalence of autoimmune diseases in the family (*P* < 0.001), and a higher proportion of preterm babies (*P* = 0.027). Regarding psychosocial factors, this group was predominantly associated with low parental education level (*P* < 0.001), single parental status (*P* < 0.001), low household income (*P* < 0.001), lack of support during pregnancy (*P* = 0.002), and lack of security for mother and child (*P* < 0.001). This group also reported maternal smoking (*P* < 0.001) and serious life events at 1 year of age (*P* = 0.002) to a greater extent than the control group (Table 1).

### Statistically independent predictors of the co-occurrence

Being male, lower household income, together with a family history of autoimmune disorders remain significant in the multiple multinomial logistic regression analyses (Table 2).

### Statistically significant predictors among the three-case groups

Serious life events during pregnancy (aOR 1.78 [95% CI 1.07–2.97]), non-Swedish father (aOR 2.39 [95% CI 1.16–4.88]), maternal age (aOR 0.95 [95% CI 0.92–0.98]), exclusive breastfeeding duration (OR 0.63 [95% CI 0.43–0.94]), and paternal education level (aOR 0.70 [95% CI 0.53–0.91]), yielded significant associations with being later diagnosed with ADHD rather than ASD. Maternal age (aOR 0.96 [95% CI 0.93–0.99]) and being male (aOR 0.67 [95% CI 0.48–0.95]), provided statistically significant discrimination between ADHD and the co-occurrence group, whereas exclusive breastfeeding duration (aOR 1.4 [95% CI 1.05–1.88]) did so between ASD and the co-occurrence group.

## Discussion

This is the first-ever prospective study considering early environmental psychosocial exposures as potential etiological factors not only for ADHD and ASD, but also for their co-occurrence.

Being male was a strong and independent predictor for later development of ADHD, ASD and their co-occurrence. Previous studies have found a male-to-female ratio of 3:1 for both ADHD^29^ and ASD^30^ while we found a ratio of 2:1. Genetic and endocrine causes have been proposed in previous studies, however, it is also possible that, due to cultural or social expectations, females may report their ADHD symptoms less frequently or as less disabling than males, thus not meeting the diagnostic criteria^31^.

Lower household income level was also independently associated with an increased risk of ADHD, ASD and their co-occurrence. Previous studies conducted in ABIS cohort and in other high-income countries, have shown marked socioeconomic inequality associated with several child health outcomes (T1D, cardiovascular risk factor and infectious diseases)^26,32,33^. On this line, previous studies found that children in families with low SES were twice as likely to have ADHD than children in high SES families^34^. Parents with ADHD diagnosis, as a background factor, could also influence both their own SES and ADHD development in their offspring^34^. According to previous studies^35^, we found that younger mothers increased the risk for ADHD in their offspring, that together with a lower education and income level could evidence socio-economic and emotional difficulties in child-rearing^36^. SES was also related to breastfeeding, as it was shown that mothers with low education levels wean earlier^37^.

In our study, longer breastfeeding was associated with lower rates of ADHD, which is in line with previous studies^15^. Breastfeeding could have a protective effect since it facilitates the intimate contact between mother and child. Furthermore, breastfeeding provides long-chain polyunsaturated fatty acids (omega 3 fatty acids, PUFA) that could affect the infant’s microbiota regulating the gut-brain-axis, important for brain development^38^. Some studies showed decreased risk for ASD also, which we could not confirm^15^. We found a positive association between smoking (at pregnancy and 1 year of age) and ADHD. Smoking per se could be a biological risk factor for ADHD, but could also indicate the exposition to a vulnerable psycho-social environment. Previous studies also reported this association^9^. Some reports have also shown that maternal stress during pregnancy is a risk factor highly associated with ASD and ADHD^8^. We found an association between serious life events during pregnancy and ADHD risk, but not ASD. Many studies have shown that prenatal exposure to maternal stress was associated with abnormalities in neurodevelopment, neurocognitive function, and cerebral processing, which lead to changes in both the hypothalamic–pituitary–adrenal axis (HPA) and the autonomic nervous system^8,39^. The underlying genetic and biological component of ASD seems to differ from the etiology of ADHD. The role of infections and the immune system in the etiology of ASD has been widely debated^40^. We consistently found that infections during pregnancy increased the risk of ASD. Accumulating evidence suggests that the immune system and abnormal immune function, including inflammation, cytokine dysregulation, and anti-brain autoantibodies, influence the trajectory of ASD^10^. Intriguingly, we found that the children of mothers with autoimmune diseases have a higher risk for ASD. Maternal hypertension, anemia, overweight, diabetes, and several other medical conditions have been also associated with an increased risk of ASD in offspring^41,42^. A meta-analysis revelated that delivery complications and cesarean section are risk factors for ASD^43^, but when differentiating between ASD and the co-occurrence group, we found that cesarean delivery seems to be a protective factor for ASD. This might be explained by avoidance of brain damage and/or hypoxia of the new-born which might otherwise have happened in a vaginal delivery. A recent systematic review, evidenced, after adjustment for familial confounding, that perinatal hypoxia and respiratory stress were consistently associated with ASD^11^. Regarding the role of genetics, a study on a large cohort of Swedish children showed that the heritability of ASD is approximately 50% and the risk of ASD increases tenfold if a sibling has the disease and twofold if a cousin is diagnosed with ASD^44^.

Regarding the ADHD/ASD co-occurrence seems to overlap the etiological pattern of ADHD, while the possible biological etiology of ASD tends to be obscured. There is evidence suggesting that autistic traits are common among children with ADHD, being ADHD a better predictor of the co-occurrence, rather than ASD^45^. A family history of autoimmune diseases was found to be a strong and independent factor for the co-occurrence (not each disorder separately), which may suggest that both disorders could share similar genetic or environmental background. Common immune-mediated diseases such as asthma and eczema have repeatedly been linked to ADHD^46–48^, similarly, ADHD has been related to autoimmune diseases, such as celiac disease, ulcerative colitis, psoriasis, ankylosing spondylitis and T1D^47,49^. It was suggested that the co-occurrence is associated with greater impairment, increased severity of psychosocial problems, and may be less responsive to standard treatments for either disorder^20^.

## Strengths and limitations

Although our results are based on a large prospective birth cohort from the general population with a follow-up for more than 20 years and the strength of merging doctor-set diagnoses of ADHD and ASD via the National Diagnosis Register, our study has some limitations. The family history of ADHD and ASD diagnoses was not possible to obtain. The lack of explicit criteria for ADHD and ASD co-occurrence could lead to variability in diagnosis and may have contributed to under-recognition or misdiagnosis of this comorbidity. Most data are based on questionnaires answered by the parents, usually the mothers, therefore there is a risk of recall bias. We have validated several data against information registered in the journals of Well Baby Clinics with good agreement, and there is no reason to believe that such registers have a systematic misdistribution that would influence our results. There are dropouts from birth to one year of age, but the remaining ABIS population is still representative of the general population. Thus, in summary, we believe that our results are robust and representative of the Swedish population.

## Conclusion

Our study includes pre and perinatal, and early environmental psychosocial exposures as etiological factors of ADHD, ASD, and their co-occurrence in a long-term prospective follow-up of a general population-based birth cohort. Observed associations suggest a genetic and biological component underlying ASD, and a larger role of environmental psychosocial factors in ADHD etiology. The co-occurrence seems to overlap the etiology of ADHD but is also influenced by a family history of autoimmune diseases. This study shed light on the factors that may confer risk for the expression and/or diagnosis of ADHD, ASD and their co-occurrence and despite these factors are not necessarily causal, may constitute important incentives for preventive measures in child health.

## Data Availability

Deidentified participant data can be shared for a specified purpose, after approval by Johnny Ludvigsson (johnny.ludvigsson@liu.se) through a signed data access agreement.
